# Development and utility of a SARS-CoV-2 pseudovirus assay for compound screening and antibody neutralization assays

**DOI:** 10.1016/j.heliyon.2024.e31392

**Published:** 2024-05-19

**Authors:** Aaron A. Manu, Irene A. Owusu, Fatima O. Oyawoye, Sylvester Languon, Ibrahim Anna Barikisu, Sylvia Tawiah-Eshun, Osbourne Quaye, Kwaku Jacob Donkor, Lily Paemka, Gloria A. Amegatcher, Prince M.D. Denyoh, Daniel Oduro-Mensah, Gordon A. Awandare, Peter K. Quashie

**Affiliations:** a West African Centre for Cell Biology of Infectious Pathogens, College of Basic and Applied Sciences, University of Ghana, Legon-Accra, Ghana; b Department of Biochemistry, Cell, and Molecular Biology, School of Biological Sciences, College of Basic and Applied Sciences, University of Ghana, Legon-Accra, Ghana; c Cellular and Molecular Biomedical Sciences Program, University of Vermont, Burlington, VT, USA; d Department of Medical Laboratory Sciences, School of Biomedical and Allied Health Sciences, Korle bu, University of Ghana, Legon, Accra, Ghana; e The Francis Crick Institute, 1 Midland Rd, London, NW1 1AT, United Kingdom

**Keywords:** SARS-CoV-2 _1_, Pseudovirus neutralization assay (PNA)_2_, pseudovirus_3_, Antiviral assay_4_, Virus inhibition_5_ COVID-19_6_ neutralizing antibodies_6_. (Min.5-Max. 8)

## Abstract

**Background:**

The highly infectious nature of SARS-CoV-2 necessitates using bio-containment facilities to study viral pathogenesis and identify potent antivirals. However, the lack of high-level bio-containment laboratories across the world has limited research efforts into SARS-CoV-2 pathogenesis and the discovery of drug candidates. Previous research has reported that non-replicating SARS-CoV-2 Spike-pseudotyped viral particles are effective tools to screen for and identify entry inhibitors and neutralizing antibodies.

**Methods:**

To generate SARS-CoV-2 pseudovirus, a lentiviral packaging plasmid p8.91, a luciferase expression plasmid pCSFLW, and SARS-CoV-2 Spike expression plasmids (Wild-type (D614G) or Delta strain) were co-transfected into HEK293 cells to produce a luciferase-expressing non-replicating pseudovirus which expresses SARS-CoV-2 spike protein on the surface. For relative quantitation, HEK293 cells expressing ACE2 (ACE2-HEK293) were infected with the pseudovirus, after which luciferase activity in the cells was measured as a relative luminescence unit. The ACE2-HEK293/Pseudovirus infection system was used to assess the antiviral effects of some compounds and plasma from COVID-19 patients to demonstrate the utility of this assay for drug discovery and neutralizing antibody screening.

**Results:**

We successfully produced lentiviral-based SARS-CoV2 pseudoviruses and ACE2-expressing HEK293 cells. The system was used to screen compounds for SARS-CoV-2 entry inhibitors and identified two compounds with potent activity against SARS-CoV-2 pseudovirus entry into cells. The assay was also employed to screen patient plasma for neutralizing antibodies against SARS-CoV-2, as a precursor to live virus screening, using successful hits.

**Significance:**

This assay is scalable and can perform medium-to high-throughput screening of antiviral compounds with neither severe biohazard risks nor the need for higher-level containment facilities. Now fully deployed in our resource-limited laboratory, this system can be applied to other highly infectious viruses by swapping out the envelope proteins in the plasmids used in pseudovirus production.

## 1 Introduction

An outbreak of the novel Coronavirus disease 2019 (COVID-19) started in Wuhan, China, in December 2019 characterized by symptoms of pneumonia and is caused by Severe Acute Respiratory Coronavirus-2 (SARS-CoV-2) [1]. This initial outbreak became a pandemic, with over 774 million confirmed cases and over 6.5 million deaths recorded as of January 14th^,^ 2024 [2].

Infection by SARS-CoV-2 causes mild to severe respiratory disease in humans, and the major transmission route is via respiratory droplets. With multiple waves of infection, each with increasing infectivity, it is imperative to have safe tools to quickly evaluate potential therapeutics and biotherapeutics, such as convalescent plasma with therapeutic potential.

SARS-CoV-2 is an RNA virus belonging to the family *Coronaviridae,* known to have the largest genome among all RNA viruses [3]. It has four structural proteins: Envelope (E), Membrane (M), Spike (S), and Nucleocapsid (N). The S protein plays an essential role in the virus's invasion of the host cell [[4], [5], [6]] -viral attachment begins with the interaction of the S protein with the host angiotensin-converting enzyme II (ACE2). Host proteases, such as furin, the transmembrane serine protease (TMPRSS), cathepsin B, and cathepsin L, act as co-facilitators [6,7].

So far, several chemotherapeutic drugs have been used in managing COVID-19 symptoms, despite limited information on whether these therapies directly affect the virus [[8], [9], [10]]. The attachment of the viral spike protein to the ACE2 receptor allows for the entry of SARS-CoV-2 into cells [6]. Thus, over-expression of ACE2 in a cell line is likely to enhance the infectivity of SARS-CoV-2 [11]. Therefore, exploiting the interaction between ACE2 and cell-surface associated Spike as a drug target will be a key step in discovering anti-SARS-CoV-2 therapy. Studies have shown that successful disruption of the spike protein-ACE2 interaction significantly inhibits SARS-CoV-2 infection [7,12,13]. Different approaches have been used to study the inhibitory effects of small molecules that potentially inhibit the interaction of the SARS-CoV-2 Spike protein with ACE2. Some approaches include computational approaches based on molecular docking and similarity analysis, *in vivo* approaches using live viruses and animal models like mice and monkeys and *in vitro* approaches using live viruses and cell lines [14,15]. These approaches are widely insufficient for initial screening and inherently pose biosafety risks. The requirement of high biosafety level facilities to reduce biosafety risks makes it difficult to apply such approaches in low and middle-income countries (LMICs) with limited resources. It is possible to circumvent these risks using non-replicating pseudoviral particles as a preliminary screening step.

This pseudoviral system requires co-transfecting cells with a lentiviral packaging plasmid, a luciferase-expressing reporter plasmid, and a plasmid-expressing the S- protein to form a SARS-CoV-2 S-pseudotyped non-replicating Luc virus in HEK293 cells [16]. The pseudotyped virus produced is capable of only a single round of infection and is safer to manipulate in the laboratory. The inclusion of a reporter protein, luciferase, enables easy quantification of coronavirus spike-mediated entry by measuring luminescence upon substrate addition. This SARS-CoV-2 pseudovirus could effectively enter HEK293 and HeLa cells transformed to express the ACE2 receptors, providing a safe and convenient method to study SARS-CoV-2 entry without the requirement of a Bio-Safety Level (BSL-3) laboratory. In this study, we developed a pseudovirus-based assay to screen compounds and plasma from SARS-CoV-2 patients for the ability to inhibit SARS-CoV-2 spike attachment and entry into mammalian cell lines. Using this assay, we identified compounds against SARS-CoV-2 entry and patients who expressed SARS-CoV-2 neutralizing antibodies. The SARS-CoV-2 pseudotype virus assay we have developed is scalable and can be used for medium to high throughput screening.

## 2 Materials and methods

### 2.1 Cell lines, plasmids, compounds, and plasma

Cell lines (HEK293 and HeLa) were obtained from American Type Culture Collection (ATCC) and cultured in Dulbecco's modified Eagle medium (DMEM) supplemented with 10 % fetal bovine serum, 2 mM l-glutamine, 1 % Penicillin-Streptomycin (Gibco, 15140148). Mammalian expression plasmids respectively expressing spike protein (pcDNA3.1-SARS-Spike, # 145031 and pcDNA3.3-SARS2-B.1.617.2, # 172320) and ACE2 receptor (pcDNA3.1-hACE2, #145033) were purchased from Addgene. The plasmids p8.91, containing the HIV packaging backbone, and pCSFLW encoding firefly luciferase were gifts from the Quaye laboratory of the Department of Biochemistry, Cell, and Molecular Biology. HEK293 cells modified to express ACE2 receptor (ACE2-HEK293) constitutively, were cultured in DMEM supplemented with 10 % fetal bovine serum, 2 mM l-glutamine, and 1000 μg/mL of Geneticin (from a 500 mg/mL stock concentration) (Gibco, 11811031). Plant-derived purported antiviral compounds were obtained from Ghana's Centre for Plant Medicine Research (CPMR), covered by a confidentiality agreement. De-identified convalescent plasma samples used for this work were retrieved from WACCBIP's COVID-19 plasma biobank. All study participants gave written informed consent. Demographic data and medical records other than vaccination of the volunteers were not included in this study. Ethical approval for the study was obtained from the Ethics Committee of the College of Basic and Applied Science (ECBAS 063/19–20) and the Ghana Health Service Ethics Committee (GHS-ERC:005/06/20). Pooled human plasma, positive for SARS-CoV-2 and calibrated to the WHO serology SARS-CoV-2 International Standard (IS) (‘Human SARS-CoV-2 Serology Standard-Lot number COVID-NS01097)’ was received as a donation from the Frederick National Laboratory for Cancer Research and was used as a positive control for seropositivity.

### 2.2 Transient expression of hACE2 in HEK293 and HeLa Cells

Using a modified calcium phosphate protocol, HEK293 or HeLa cells were transfected with the human ACE2 plasmid, *pcDNA3.1-hACE2* (*hACE2*). Briefly, 300000 cells were seeded in a 10 cm dish and incubated overnight at 37 °C with 5 % CO_2_. After 21 h before transfection, the media was replenished, and cells were kept under the same conditions for 3 more hours before transfection. To initiate transfection into HEK293 cells, *hACE* plasmid DNA (3, 5, or 8 μg), and 12.2 μL of 2 M CaCl_2_ were added to a sterile microfuge tube. Nuclease-free water will be added to a total volume of 100 μL, then mixed gently (for transfection of HeLa Cells, 5 μg of hACE plasmid was used). The mixture was added dropwise to another sterile tube containing 100 μL of 2x HEPES Buffered Saline (HBS), then incubated at room temperature for 30 min. The transfection mixture was gently mixed at 10-min intervals by tapping the base of the tube. The resulting plasmid-calcium phosphate complex in solution was added dropwise to the seeded cells. Cells were harvested 24 h post-transfections, and ACE2 expression was confirmed by Dot blot analysis, then used for pseudovirus infection experiments.

### *2.3* *Production of ACE2-HEK293 which stably expresses* hACE2

Neomycin resistance encoded within the *pcDNA3.1-hACE2* plasmid was used to select cells that expressed hACE2 over multiple passages. A kill curve was used to determine the required antibiotic (geneticin/G418) concentration to kill all non-transfected cells within two weeks. Briefly, cells were incubated in media supplemented with increasing concentrations of G418 for 2 weeks. Cell viability, after two weeks, was determined microscopically using a haemocytometer and trypan blue staining to count viable and dead cells. On this basis, 1000 μg/mL of G418 was found safe for subsequent use. After transfection, as described above, cells were cultured for two weeks, during which media was changed every two days with fresh media containing 1000 μg/mL geneticin. After two weeks, geneticin concentration was reduced to 500 μg/mL, and eventually, the cells were passaged in 100 μg/mL Geneticin. After the expression of *ACE2-HEK293* cells was confirmed, cells were frozen in a freezing medium and then stored in liquid nitrogen. Expression of ACE2 was verified in freshly thawed cells using a Dot blot assay.

### 2.4 Dot blot to detect expression of ACE2 in cells after transfection

To verify the expression of the ACE2 receptor, ACE2-HEK293 cells were lysed using the freeze-thaw method in the presence of a protease inhibitor (SIGMA, SCLF5528). Briefly, frozen ACE2-HEK293 cells were thawed to 37 °C, and then spiked with 1X protease inhibitor. The cells are then frozen again at −80 °C for 10 min and then thawed to 37 °C again. This cycle (except the spiking with protease inhibitor) was repeated 5 times for effective lysis. The lysate (2–10 μL) was spotted on a nitrocellulose membrane and allowed to air dry on a clean bench. The membrane was then blocked with 5 % BSA in Tris: 20 mM, NaCl: 150 mM, Tween® 20 detergent: 0.1 % (w/v) (TBS-T) for 30 min at room temperature. The membrane was probed with rabbit polyclonal (1:1000 dilution) anti-hACE2 (Abcam, GR3333640-16) for 1 h at room temperature. The membrane was then washed thrice using TBS-T for 10 min at room temperature. To detect bound ACE2, the membrane was incubated with a 1:1000 dilution of goat anti-rabbit IgG HRP-conjugated antibody (Abcam, GR3299244-1) for 1 h at room temperature. The membrane was then washed three times with 1X TBS-T for 10 min at room temperature, followed by a final wash of 1X TBS for 10 min at room temperature. Following manufacturer protocol, a mixture of enhanced chemiluminescence reagent (Protein biology, VB296357) and hydrogen peroxide was prepared in a 1:1 ratio and poured on the blot immediately before imaging. Blots were visualized using the Amersham™ Imager 600.

### 2.5 Production of SARS-CoV-2 S pseudoviruses

**Fig. 1:**
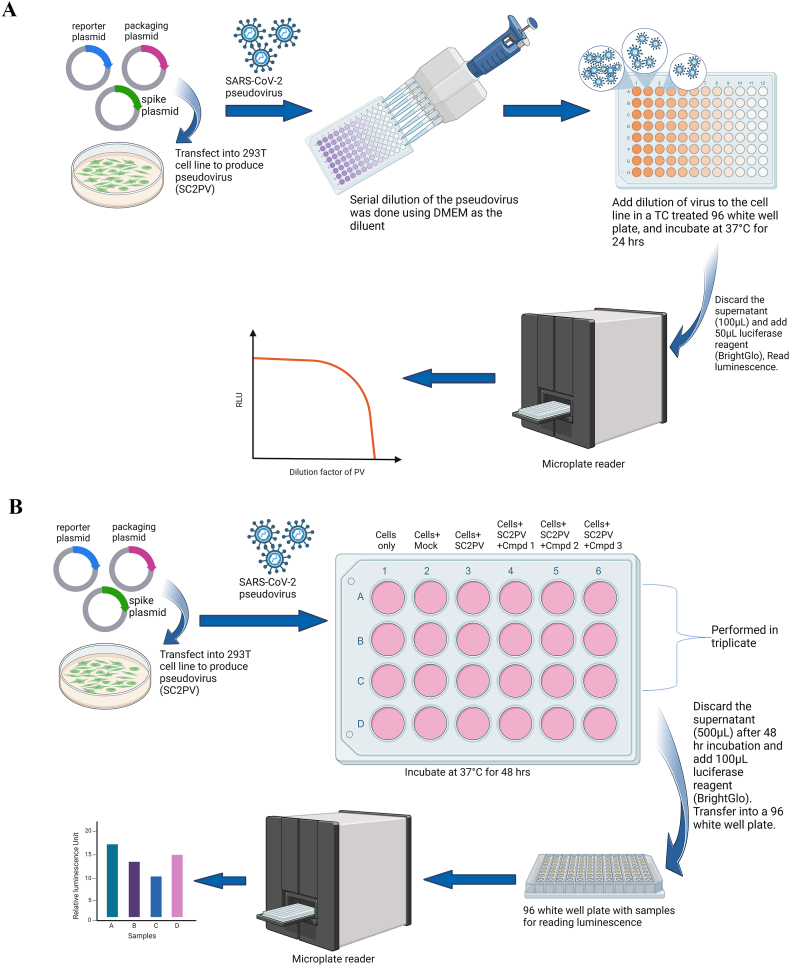

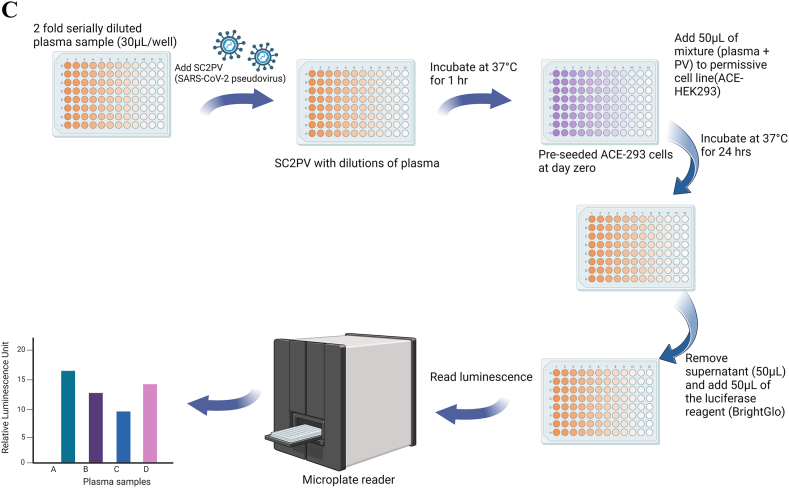
Pseudovirus production and utility to screen antiviral compounds and neutralizing plasma. Diagram showing workflow for pseudovirus production, (A) its usage to screen compounds for antiviral effects (B), and usage to screen plasma for neutralizing antibodies (C). SC2PV = SARS-CoV-2 pseudovirus, Cmpd = compound. This image was created by the author using BioRender.com (accessed on: February 7th, 2023 for “A” and February 22nd, 2023 for “B” and “C”).

Using the calcium phosphate transfection method described above, a 3rd generation three-plasmid lentiviral system was used to produce SARS-CoV-2 Spike expressing pseudovirus (SC2_PV_). Briefly, 24 h before transfection, 5 000000 HEK293 cells were seeded into a 10 cm tissue culture dish and incubated at 37 °C with 5 % CO_2_. Media was replenished 3 h before transfection of the plasmids. A summary of the workflow for pseudovirus production and titration is illustrated in Fig. 1A. The three plasmids were co-transfected into the HEK293 cells using the calcium phosphate precipitation method as described above. A 100 μL mixture (solution A), comprising 5 μg of spike plasmid,3 μg of reporter/luciferase plasmid (PCSFLW), 2 μg of Gag pol packaging system plasmid (p8.91), 12.2 μL of 2 M CaCl_2_ and nuclease-free water. Solution A was added dropwise to 100 μL of 2X HBS and incubated at room temperature for 30 min with gentle mixing at 10-min intervals. The resulting solution was dropped wisely and added to the cells in the dish that had been replenished with fresh medium 3 h earlier. The medium was changed after 24 h and pseudovirus in the supernatant was harvested after 48 h and 72 h post-transfection and then filtered with a 0.45um filter.

To quantify the PV produced, the inoculum was serially diluted (1:2) eleven times in a round-bottom 96-well plate in duplicate. The dilutions were then transferred onto ACE2-HEK293 cells, pre-seeded in a white 96-well plate (136101) at 20000 cells per well. The infected cells were incubated at 37 °C in the presence of 5 % CO_2_ for 24 h. Bright Glo luciferase reagent was used to measure luciferase activity (in RLU/mL) as a measure of pseudoviral titre. A working of 1,200,000 RLU/mL cut-off was selected by plotting a graph of RLU/mL against the dilution factor.

### 2.6 Screening for SARS-CoV-2 entry inhibitors

Freshly thawed ACE2-HEK293 cells were seeded in a 6-well tissue culture plate at 3 × 10^5^ cells per well and incubated at 37 °C with 5 % CO_2_. After 24 h of seeding, cells were replenished with a warm culture medium with or without pseudovirus (SC2_PV_) infection and compound treatment or an equivalent concentration of DMSO. Cell treatment conditions included DMSO (cells only), lentivirus backbone only (mock infection), SC2_PV_ only (represents infection control), SC2_PV_ with compound 1, and SC2_PV_ with compound 2, as illustrated in Fig. 1B. Before treatment, 100 μL of SC2_PV_ was mixed with either compounds or DMSO diluted in 400 μL of the warm medium in a microtube bringing it to a volume of 500 μL. The old medium was aspirated off the cells and replaced with the 500 μL mixture in the tubes. For SC2_PV_ only and mock treatments, 100 μL of SC2_PV_ or lentivirus backbone was mixed with 400 μL medium before transferring to their respective wells. Treated cells were incubated at 37 °C with 5 % CO_2_ for 48 h. Luciferase activity, which reflects pseudovirus infectivity-was determined using Bright Glo reagent (Promega and E2650) following the manufacturer's instructions. Luminescence was read in relative luminescence unit (RLU) using the GloMax® Discover Microplate Reader (Promega-GM3000). The experiment was done in four replicates. To calculate the SC2_PV_ infectivity after each compound treatment, the corresponding RLU was divided by the average RLU of the infection control and expressed as a percentage.

### 2.7 Using assay to screen for neutralizing COVID-19 convalescent plasma

The pseudovirus system was used to determine the neutralizing titers in the plasma of COVID-19 patients (Fig. 1C). Plasma samples were heat-inactivated at 56 °C for 30 min before serial dilutions. The samples were first diluted 1 in 10 before subsequent 2-fold serial dilutions to a 30 μL final volume in a round-bottom 96-well plate. Pseudovirus stock was also diluted to obtain 1,200,000 RLU/mL. 30 μL of the diluted pseudovirus was added to the plasma dilutions in the plate, making the total volume 60 μL. The plate was spun down at 500 rpm for 30 s and incubated for 1 h at 37 °C (5 % CO_2_). About 2 min before the end of the 1-h incubation, ACE-293 cells seeded 18 h earlier in white 96-well plates at 20,000 cells per well were removed from the incubator to aspirate and discard the medium. After the incubation, 50 μL of the plasma/pseudovirus mixture was transferred from the round-bottom 96-well plate onto the ACE-293 cells in the white plate and incubated at 37 °C with 5 % CO_2_ for 24 h to allow infection. After infection, the medium was aspirated off the cells in the plate, and 50 μL of a 1:1 mixture of Bright-Glo (Promega) luciferase reagent and DMEM was added to the cells in the dark. The plate was incubated in the dark with slow rocking at room temperature for 3 min before transferring to a plate reader to measure luminescence.

### 2.8 Statistical analysis

Data were analysed using GraphPad Prism software (Version 9). Comparison of infectivity was done using one-way ANOVA and paired *t*-test. A p-value of <0.05 was considered statistically significant.

## 3 Results

### 3.1 Human ACE2 are expressed transiently or constitutively in HEK293 and HeLa cells

**Fig. 2:**
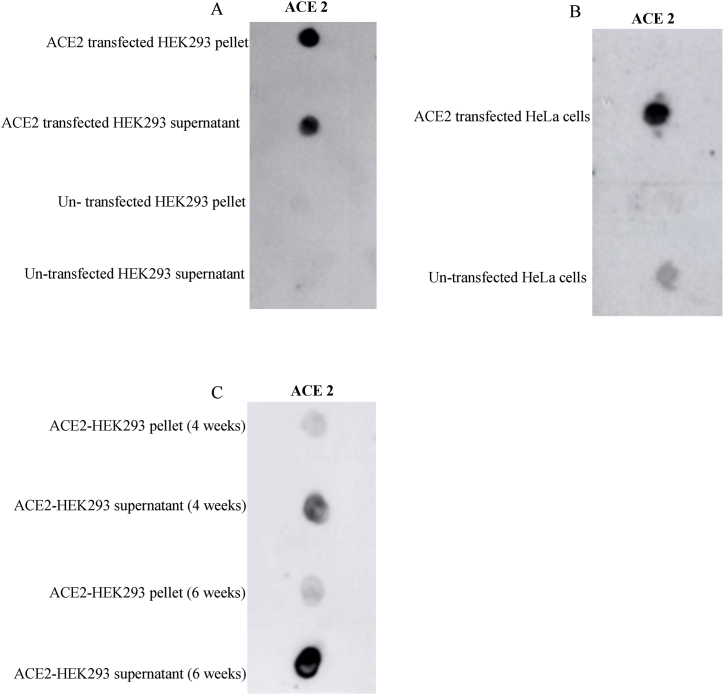
Dot blots showing the successful expression of ACE2 receptors in HEK293 and HeLa cells. Transient expression of human ACE2 is shown in the dot blot for HEK293 (A) and HeLa (B) cells after transfection with ACE2 plasmids. Constitutive expression of human ACE2 in the supernatant and pellet of HEK293 cells at 4 and 6 weeks in culture is also demonstrated (C).A.

HEK293 and HeLa cells, commonly available in the lab, respectively express low levels and no detectable ACE2 receptors on the cells. To make these cells permissive to *in vitro* SARS-CoV-2 infection, the ACE2 receptor was expressed in these cells following transfection with the ACE2 expression plasmid. The dot blot confirmed that after transfection, there was successful transient over-expression of ACE2 in HEK293 (Fig. 2A) and HeLa (Fig. 2B) cells compared to the un-transfected ones. The ACE2 receptor was also successfully stably expressed in transfected HEK 293 cells in culture post-transfection after 4 and 6 weeks (Fig. 2C).

### 3.2 SARS-CoV-2 spike pseudovirus production using a three-plasmid system, and pseudovirus infection increases with ACE2 expression

**Fig. 3:**
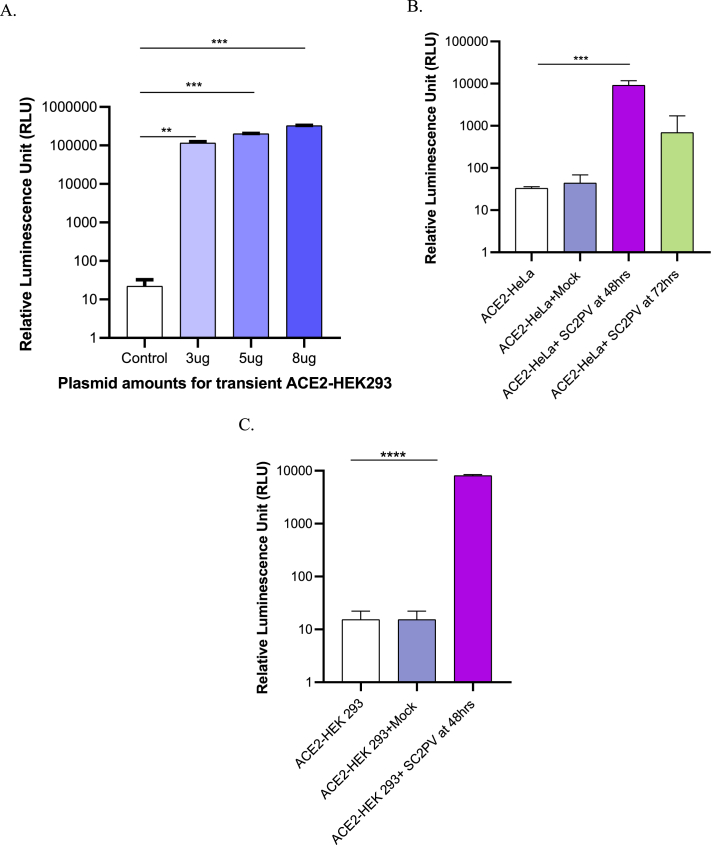
Optimizing the pseudovirus system for ACE2 expression and pseudovirus production time. The bar charts show 24-h infectivity of SARS-CoV-2 spike protein pseudovirus (SC2_PV_) in HEK293 and HeLa cell lines. **(A)** The graph shows increased SC2PV infectivity with an increase in ACE2 plasmid concentration when transfected into HEK293 cells. Control is native cells without ACE2 transfection; x μg represents the concentration of ACE2 plasmid transfected to cells. **(B)** Graph showing different levels of infectivity of SC2_PV_ produced at 48 and 72 h post-transfection in HeLa cells expressing ACE2. **(C)** Graph showing the infection of ACE2-HEK 293 with pseudovirus harvested at the optimum time (48 h). Controls include cells without SC2_PV_ infection (black bars) and cells with pseudovirus with only lentivirus backbone without the SARS-CoV-2 spike protein (mock). Data are mean with SD. **p < 0.01; ***p < 0.001; ****p < 0.0001 - using One-Way ANOVA and *t*-test. Each experiment was replicated at least three times.

A single-cycle luciferase-expressing non-replicating lentivirus-based virus-like particle expressing SARS-CoV-2 spike protein was produced after simultaneously transfecting three plasmids to cells. Pseudoviral infectivity was correlated to amounts of ACE2 plasmid used in the transfection (Fig. 3A), implying the importance of ACE2 expression on infectivity. For subsequent assays, 5 μg of pcDNA 3.1 hACE2 was used for the transfections. Luciferase bioluminescence was measured in infected cells to test the infectivity of the pseudoviruses at different time points on HeLa cells expressing ACE2. Pseudovirus infectivity was highest after 48 h of infection compared to 72 h, and ACE2 expression in cells significantly enhanced infectivity (p = 0.0182) in HeLa (Fig. 3B). Also, ACE2-HEK293 cells were tested for infectivity of the pseudoviruses at a 48-h time point; a luciferase test was run on infected cells. There was a significant increase in pseudovirus infectivity after 48 h compared to 72 h, and ACE2 expression in cells significantly enhanced infectivity (p = 0.0182) in HeLa (Fig. 3B). Pseudovirus harvested at 48 h was used to infect ACE2-HEK293 cells, and there was a significant difference between cells infected with SARC-CoV-2 Pseudovirus compared to the mock (Fig. 3C).

### 3.3 Using the assay to evaluate antiviral compounds

After successfully producing the pseudoviruses (SC2_PV_) and ectopically expressing hACE2 in cells, the functionality of the hACE2-expression/pseudovirus system was determined. The system was used to test the activity of five compounds in inhibiting pseudovirus entry/infection (Table 1). The results showed that two out of the five compounds tested (compounds 2 and 5) significantly reduced SC2_PV_ infectivity by 99.87 % and 78 %, respectively compared to the SC2_PV_ infection control set at 100 % infectivity. Decreased SC2_PV_ infectivity implies inhibition of infection. While treatment with compounds 1 and 4 showed comparable SC2_PV_ infectivity with the untreated SC2_PV_, compound 3 significantly increased SC2_PV_ infectivity by 36 %. Increased PV infectivity implies a possible agonistic effect.

**Table 1:** Showing potential compounds that can inhibit or enhance SC2PV entry in HEK293 cells after screening with the pseudoviral assay. ns = not significant.

							SC2_PV_ %Inhibition (100-infectivity)	SC2_PV_ %Agonism (infectivity-100)
	Luminescence (RLU)				Average luminescence (RLU)	Average SC2_PV_ % infectivity (P-value)		
	1	2	3	4				
Compound 1	46340	52820	41200	41290	45412.5	105.25 (ns)	−5.25	5.25
Compound 2	63	63	50	40	54	0.13 (<0.0001)	**99.87**	−99.87
Compound 3	66300	67850	50380	50150	58670	136 (<0.01)	−36	**36**
Compound 4	42040	49080	39400	55690	46552.5	107.75 (ns)	−7.75	7.75
Compound 5	10150	11830	7906	8103	9497.25	22 (<0.0001)	**78**	−78
Average SC2_PV_ infection control	**43125 RLU**							
Average cell control	**63 RLU**							

### 3.4 Using the assay to evaluate virus neutralizing activity of COVID-19 convalescent plasma

**Fig. 4:**
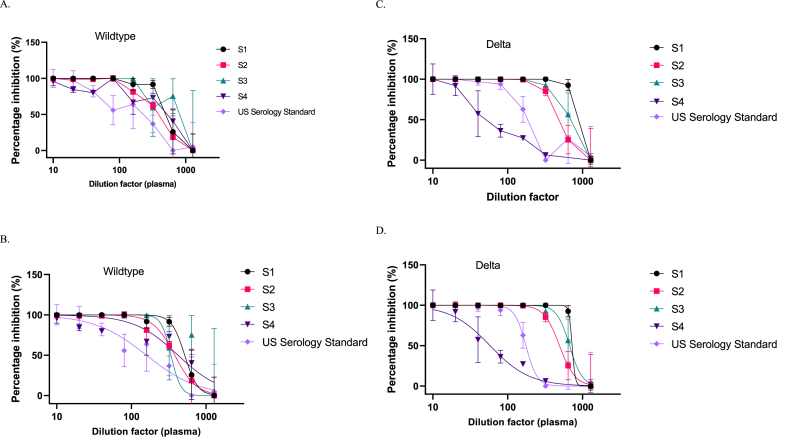
SARS-CoV-2 pseudovirus neutralization by plasma from COVID-19 patients. In four patient samples, S1–S4 showed varying degrees of inhibiting the wildtype spike pseudovirus (A & B) and the Delta variant spike pseudovirus (C & D). B and D are best-fit curves for A and C, respectively. A US serology secondary standard was used as positive control.

One of the most valuable uses of pseudoviral assays is to detect patient plasma with neutralizing activity (Fig. 4 A-D, and Table 2). Thus, the neutralizing activity of four patient plasma against D164G and Delta pseudoviruses was evaluated. The inhibition capacities of different dilutions of the patient plasma samples were determined against wildtype D164G spike pseudovirus (Fig. 4A and B) and the Delta variant pseudovirus (Fig. 4C and D). These dilutions against pseudovirus inhibition in Fig. 4A and C were used to calculate the 50 % neutralizing titers presented in Table 2. Plasma sample 1 (S1) was obtained at the peak of the Omicron wave, S2 and S3 were obtained during the Delta wave, and S4 was confirmed to be an Omicron infection (Table 2). S3 and S4 were from vaccinated individuals. All plasma samples showed higher neutralizing activity than the serology standard for the Wuhan pseudovirus. Samples S1, S2, and S3 showed strong neutralizing activity against Delta. Plasma S4, from an Omicron-infected patient, showed poor neutralizing activity against Delta, as can be expected, since Omicron evolved to evade Delta neutralization [17,18].

**Table 2:** Represents the neutralizing titers of each plasma sample at 50 % inhibition. The data in the table shows plasma samples' measured ability to neutralize the SARS-CoV-2 virus particles. It also provides the circulating variants as of the time of sample collection and some data on the vaccination status and occupation of the participants.

Samples (plasma)	Major Circulating Variants	Vaccination status	Neutralizing titer 50 (NT 50)	
			D614G	Delta
S1	Omicron	No	516.9 ± 39.8	827.3 ± 35.1
S2	Delta	No	380.2 ± 39.9	461.2 ± 40.1
S3	Delta	Yes	338.9 ± 35.4	779.6 ± 36.0
S4	Omicron* (sequence confirmed)	Yes	420.8 ± 33.0	58.13 ± 39.3
US Serology Standard			158.6 ± 38.6	173.5 ± 43.9

## 4 Discussion

To facilitate the rapid and safe screening of antiviral compounds, pseudoviruses or virus-like particles are frequently preferred for prescreening prior to live viruses. Lentiviral vectors can be pseudotyped with viral envelope glycoproteins that modulate cellular tropism and cell-entry properties [19]. Pseudovirus-based assays have been widely used to study cellular tropism, receptor recognition, and viral inhibitors, as well as to evaluate neutralizing antibodies [20]. Here, we generated permissive cell lines and SARS-CoV-2 spike pseudoviruses to identify SARS-CoV-2 entry-inhibitors and convalescent plasma with neutralizing activity.

This pseudovirus system which expressed different spike variants, was used for antiviral drug screening and neutralizing antibody screening in two cell lines. This implies that different pseudoviral particles apart from SARS-CoV-2 can be adapted to this system. Our lentivirus system presents a shorter assay time between pseudovirus production and its utility. It takes less than 72 h to prepare a fresh batch of pseudovirus and less than 48 h to infect cells and read out results after drug treatment or antibody neutralization. A maximum of 4–5 days is enough to produce pseudovirus and test for anti-viral agents such as drugs or antibodies, and a much shorter time of 1–2 days when a pseudovirus stock is already available. The functionality of the assay allowed the identification of two putative entry inhibitors (Compound 2 and 5) and a putative entry agonist (Compound 3). We also confirmed neutralizing activity in SARS-CoV-2 convalescent plasma and negative activity in SARS-CoV-2 negative plasma. The use of lentiviral pseudoviral systems for SARS-CoV-2 is well established, and as such extensive validation was not carried out. However, unlike similar studies that use commercial lipofection methods [21], this study used a cheaper calcium phosphate transfection method to express ACE2 on the HEK293 and HeLa cell lines and produce the pseudoviruses. ACE2 was successfully expressed on both cell lines.

This study did not evaluate the viability of the produced pseudoviruses after several freeze-thaws, and we therefore recommend freezing and storing single-use aliquots of pseudoviruses at −80 °C. Additionally, the pseudovirus dosage curve and the detection limit were not determined. We did not show direct evidence of the accuracy, precision, and specificity of this method, the ability of this assay to determine differential antiviral effects of the compounds tested makes it a good tool for the initial screening of antivirals. Similarly, the ability of the assay to confirm the neutralizing activity of previously tested positive plasma confirms some level of accuracy for a screening tool. Any subsequent use for semi-quantitative methods will need to be accompanied by generating a calibration curve. Another limitation is that we were unable to carry out a live virus experiment to validate the pseudovirus data because we are still in the process of setting up live virus assays.

In conclusion, we have created stable ACE2-expressing HEK293 cells and showed that ACE2 expression made them highly susceptible to infection by SARS-CoV-2. We have optimized a 3rd generation lentiviral system to consistently produce active SARS-CoV-2 pseudoviral particles and confirmed the utility of these pseudoviral particles to be inhibited by antiviral agents. We have also confirmed the utility of this ACE-HEK293/Pseudoviral system to variants be used to screen for plasma with neutralizing activity against different pseudovirus variants. This assay also has the utility of compound screening for antiviral drug discovery. Since we ectopically expressed ACE2 on cells, this system can be modified to test the effect of host factors like different ACE2 polymorphisms on virus infectivity. Thus, our system is helpful for testing antiviral agents and can be applied to study virus-host interactions to evaluate both host (receptor polymorphisms) and virus (variants) factors that contribute to optimum infections.

## Funding

This work was supported, in part, by the Crick African Network (CAN/A00004/1 to PKQ) which receives its funding from the UK's Global Challenges Research Fund (MR/P028071/1), and by the 10.13039/100010438Francis Crick Institute which receives its core funding from 10.13039/501100000289Cancer Research UK (FC1001647), the 10.13039/100007472UK 10.13039/501100000265Medical Research Council (FC1001647), and the 10.13039/100010269Wellcome Trust (FC1001647). This publication is also based on research funded by the 10.13039/100000865Bill & Melinda Gates Foundation (INV-036307). FOO was supported by a WACCBIP-10.13039/100004421World Bank ACE Masters fellowship (WACCBIP + NCDs: Awandare). For Open Access, the author has applied a CC BY public copyright license to any Author Accepted Manuscript version arising from this submission. The findings and conclusions contained within are those of the authors and do not necessarily reflect the positions or policies of the Funders.

## CRediT authorship contribution statement

**Aaron A. Manu:** Writing – review & editing, Writing – original draft, Methodology, Investigation, Formal analysis. **Irene A. Owusu:** Writing – review & editing, Writing – original draft, Investigation. **Fatima O. Oyawoye:** Writing – original draft, Investigation. **Sylvester Languon:** Writing – review & editing, Investigation. **Ibrahim Anna Barikisu:** Investigation. **Sylvia Tawiah-Eshun:** Investigation. **Osbourne Quaye:** Resources. **Kwaku Jacob Donkor:** Investigation. **Lily Paemka:** Resources. **Gloria A. Amegatcher:** Investigation. **Prince M.D. Denyoh:** Investigation. **Daniel Oduro-Mensah:** Supervision, Conceptualization. **Gordon A. Awandare:** Funding acquisition, Conceptualization. **Peter K. Quashie:** Writing – review & editing, Supervision, Funding acquisition, Conceptualization.

## Declaration of competing interest

The authors declare that they have no known competing financial interests or personal relationships that could have appeared to influence the work reported in this paper.
